# Molecular Characterization of *Giardia duodenalis* in Children in Kenya

**DOI:** 10.1186/s12879-016-1436-z

**Published:** 2016-03-22

**Authors:** C. Mbae, E. Mulinge, F. Guleid, J. Wainaina, A. Waruru, Z. K. Njiru, S. Kariuki

**Affiliations:** Centre for Microbiological Research, KEMRI, P.O Box 19464–00202, Nairobi, Kenya; International Livestock Research Institute, Naivasha Rd, P.O Box 30709, Nairobi, Kenya; Kenya Medical Research Institute, P. O Box 58540–00200, Nairobi, Kenya; Murdoch University, School of Health Professions, Peel Campus, Mandurah, WA 6210 Australia

**Keywords:** *Giardia*, Informal settlements, Children, Kenya, Genotyping, Subtyping

## Abstract

**Background:**

*Giardia duodenalis* is an important intestinal protozoan in humans worldwide with high infection rates occurring in densely populated and low resource settings. The parasite has been recorded to cause diarrhea in children. This study was carried out to identify *G. duodenalis* assemblages and sub-assemblages in children presenting with diarrhea in Kenya.

**Methods:**

A total of 2112 faecal samples were collected from children aged ≤5 years and screened for the presence of *Giardia* cysts using microscopy. A total of 96 (4.5 %) samples were identified as *Giardia* positive samples and were genotyped using *glutamate dehydrogenase (gdh)*, *triose phosphate isomerase (tpi)* and *β-giardin* loci.

**Results:**

The three markers successfully genotyped 72 isolates and grouped 2 (1.4) isolates as Assemblage A, 64 (88.9) as Assemblage B and 7 (9.7 %) consisted of mixed infections with assemblage A and B. A further analysis of 50 isolates using *GDH* Polymerase Chain Reaction and Restriction Fragment Length Polymorphism (PCR-RFLP) categorized 2 assemblage A isolates as sub-assemblage AII while 6 and 14 assemblage B isolates were categorized into sub-assemblage BIII and BIV respectively. A mixed infection with sub-assemblage BIII and BIV was recorded in 28 isolates. Over half (55.6 %) of *Giardia* infections were recorded among the children between 13 to 48 months old.

**Conclusion:**

This paper reports the first data on the assemblages and sub-assemblages of *Giardia duodenalis* in children representing with diarrhea in Kenya.

## Background

*Giardia duodenalis* is a flagellated protozoan infecting humans and a wide range of animals worldwide, mainly transmitted through food and water contaminated with cysts [1, 2]. In Asia, Africa, and Latin America, approximately 200 million people have symptomatic giardiasis with some 500,000 new cases being reported each year [3]. Previous studies on *G. duodenalis* had shown that the species comprises eight distinct genetic groups designated as assemblages A to H and which differ on the basis of host occurrence and genomic mutations [4, 5]. All the assemblages have similar morphology and are indistinguishable using microscopy.

The genotyping of a large number of human *Giardia* isolates from different parts of the world revealed that humans are mainly infected with assemblage A or B with assemblage B being the most common [5]. Moreover these assemblages are found in numerous species of mammals and hence they are considered zoonotic. The assemblages C to H appear to be restricted to animals and are host specific, however occasionally assemblage C and D [6, 7], E [8] and F [9] have been reported in humans.

Three sub-assemblages have been identified within Assemblage A and namely AI, AII and AIII [5, 10]. The sub-assemblage AI is zoonotic, while subtype AII predominantly occurs in humans [11] and subtype AIII has solely been identified in animals (mainly wild ungulates) [12]. Within assemblage B, sub-assemblages BIII and BIV have been identified [13] and detected in humans, companion animals and wildlife. Studies searching for differences in clinical symptoms between people infected with assemblages A and B have reported varying results. Some studies reported a strong association between intermittent diarrhoea and assemblage A infection while persistent diarrhoea was strongly associated with assemblage B infection, while in others, children infected with assemblage A were more likely to be symptomatic compared with those infected with assemblage B [14, 15].

The use of multi-locus genotyping approach using *β-giardin*, *GDH, Tpi,* SSU rRNA, *ef1*α, and variant surface protein [*vsp*] genes), is the preferred method for studying genetic variability in *G. duodenalis* from different hosts [5, 6]. Moreover the use of primers based on *Tpi* marker detected more mixed infections with assemblage A and B than when general PCR primers were used [16, 17]. In this paper, we report the detection and genetic variability of *G. duodenalis* in Human Immunodeficiency Virus infected and/or uninfected children presenting with diarrhoea in outpatient clinics at Mukuru informal settlement on the outskirt of Nairobi, Kenya and those admitted at the Paediatric ward at the Mbagathi district hospital in Nairobi.

## Methods

### Sampling and microscopy

Approximately 20 g of stool sample were collected from each child aged ≤5 years and who presented with diarrhea at the participating outpatient clinics and hospital. A total of 2112 faecal samples were collected and screened for the presence of *Giardia* cysts using microscopy. The stool was examined macroscopically for consistency, mucus and blood, and microscopically for the presence of ova, larvae, trophozoites or cysts of intestinal and extra-intestinal parasites through the formal-ether concentration method as described by Cheesebrough, (2005). Results of the parasitological survey are detailed in previously published study [18]. A total of 98(4.6 %) samples were identified as *Giardia* positive.

### DNA extraction

A total of 98 samples identified positive for *Giardia* through microscopy were processed for extraction of genomic DNA using QiAmp® DNA stool Mini kit (Qiagen, Crawley, West Sussex, UK) and following the manufacturers protocol. The resulting DNA was aliquoted and stored at −20 °C until further needed.

### Amplification and restriction digestion of the *GDH* gene

A fragment of the GDH gene of *Giardia* (432 bp) was amplified by seminested PCR using the primers GDHeF, GDHiR and GDHiF as previously described (Read et al. [6]). The resulting products were visualized on 1.5 % agarose gels stained with ethidium bromide. The resulting secondary PCR products were analysed by restriction (RFLP) through digestion with the restriction endonucleases *Nla* IV and *Rsa*I separately to distinguish sub-assembages AI, AII, BIII and BIV [6]. The resulting profiles were visualized on 2 % high resolution grade agarose stained with ethidium bromide.

### Amplification of *Tpi* gene

A fragment of approximately 605 bp of the *tpi* gene was obtained using the external primers AL3543 and AL3546, and internal primers AL3544 and AL3545 from primary PCR [19]. This was followed by two separate specific nested PCRs for assemblage A [16] that gave expected amplicons of 373 bp and assemblage B [17] that showed amplicons of approximately 400 bp.

### Amplification and sequencing of *β-giardin* gene

The primary fragment of *Giardia β-giardin* gene was amplified as described [10] and followed by the amplification of a secondary fragment of 511 bp using a nested PCR. The resulting amplicon were identified using a 2 % high resolution grade agarose stained with ethidium bromide.

The reaction mixtures containing the correct size fragment of 511 bp were purified using QIAquick PCR purification kit (Qiagen GmbH, Hilden, Germany) following the manufacturer’s protocol. The resulting DNA was quantified using Nanodrop and the eluates that had concentrations of 50 ng/μl were prepared for sequencing. A total of 41 PCR products were sequenced in both directions using forward primer bGiarF 5′-GAACGAGATCGAGGTCCG-3′and reverse primer bGiarR 5′CTCGACGAGCTTCGTTGTT-3′ [12]. The DNA sequencing was carried out using the ABI Big Dye terminator sequencing kit. All pairs of sequences obtained were edited and consensus sequence generated using CLC DNA workbench 6.1 (CLC Bio, www.clcbio.com). Each consensus sequence from individual isolates was used for the identification of *β-giardin* assemblages and sub-assemblages.

### Phylogenetic analysis

The resulting sequences were blasted using the basic local alignment search tool (BLAST) (http://blast.ncbi.nlm.nih.gov/Blast.cgi) to determine genetic relatedness of individual assemblages with sequences in GenBank. Multiple sequence alignment of the representative *Giardia* isolates and reference sequences of various assemblages was done using ClustalX 2.1 [19]. To assess the extent of genetic diversity of *Giardia* species in samples and their evolutionary relationships to other *Giardia* assemblages and sub-assemblages, a phylogenetic analysis was carried out using the software package MEGA version 5.05 [20]. Representative *β*-giardin gene sequences from each major *G. duodenalis* assemblages AI, AII, BIII, BIV and D with GenBank accession numbers X85958, AY072723, AY072725, AY072726, AY072727 and AY545648 were used as reference. *Giardia ardeae* (GenBank accession number AF069060) was used as out-group.

### Ethics statement

The study was approved by the Kenya National Ethical Review Committee (SSC No. 1579). All parents and/or guardians of participating children were informed of the study objectives and voluntary written consent was sought and obtained before inclusion. A copy of the signed consent was filed and stored in password protected cabinets at KEMRI.

## Results

### Multi-locus PCR

Microscopy showed 98 stool samples to be positive for *Giardia* of which 80(83 %) were positive using the PCR tests targeting *GDH*, *Tpi* and β *giardin* loci while sixteen were negative. The expected 432 bp *GDH* gene fragment was amplified in 73/96 (76 %) samples, however only 50 samples gave strong PCR products for RFLP analysis. The RFLP classified two isolates as assemblage A and 48 as assemblage B (Table 1). The Assemblage A DNA showed RFLP patterns of 70, 80, 90 120 bp, typical for sub-assemblage AII with the *NlaIV* enzyme. Among the 48 assemblage B isolates, six were identified as sub-assemblage BIII, 14 as sub-assemblage BIV and 28 showed patterns of both BIII and BIV (Table 2). The *Tpi* gene was amplified in 63 samples of which one was grouped as assemblage A, 56 as assemblage B and six DNA samples showed presence of both mixed assemblage A and B.

**Table 1:** The distribution of *Giardia* assemblages A and B in children with diarrhea

				Assemblages	
Patient characteristic	Category	Total (%)	A (%)	B (%)	A&B^a^ (%)
Gender	Male	44(61.1)	1(100)	39(60.9)	4(57.1)
	Female	28(38.9)	0(0)	25(39.1)	3(42.9)
Patient type	Out-patient	67(93.1)	1(100)	59(92.2)	7(100)
	In-patient	5(6.9)	0(0)	5(7.8)	0(0)
Age group	0–12 months	5(6.9)	0(0)	5(7.8)	0(0)
	13–24 months	21(29.2)	1(100)	18(28.1)	2(28.6)
	25–36 months	19(26.4)	0(0)	17(26.6)	2(28.6)
	37–48 months	16(22.2)	0(0)	13(20.3)	3(42.9)
	49–60 months	11(15.3)	0(0)	11(17.2)	0(0)
HIV status	Positive	7(9.9)	0(0)	7(11.1)	0(0)
	Negative	64(90.1)	1(100)	56(88.9)	7(100)

^a^Number of patients with co-infection of A and B assemblage

Distribution of *Giardia* assemblages A, B and mixed infections with A&B in relation to gender, age, patient type, HIV status and seasons, and distribution of the assemblages among children presenting with different clinical symptoms. The assemblages were identified through PCR-RFLP targeting *GDH* gene

**Table 2:** The distribution of *Giardia* sub-assemblages in children representing with diarrhea

			*Giardia* sub-Assemblagescpe			
Patient characteristics	Category	Total	AII&BIII	BIII	BIII&BIV	BIV
Gender	Male	34	1	4	20	9
	Female	16	1	2	8	5
Age group	0 to 12 months	3	0	0	1	2
	13 to 24 months	18	0	3	10	5
	25 to 36 months	14	1	0	11	2
	37 to 48 months	6	1	2	2	1
	49 to 60 months	9	0	1	4	4
HIV status	Positive	4	0	2	0	2
	Negative	46	2	4	28	12
Patient type	Out-patient	46	2	6	27	11
	In-patient	4	0	0	1	3
Acute diarrhea	No	18	1	1	14	2
	Yes	32	2	5	14	12
Chronic diarrhea	5	43	2	5	23	13
	12	7	0	1	12	1
Vomiting	No	22	2	2	16	6
	Yes	28	0	4	16	8
Abdominal pain	No	20	2	2	10	6
	Yes	30	0	4	18	8
Fever	No	25	4	14	6	6
	Yes	25	2	14	8	8
Season	Dry season	24	5	13	5	5
	Wet season	26	1	15	9	9

Distribution of *Giardia* sub-assemblages in relation to gender, age, patient type, HIV status and seasons, and distribution of the sub-assemblages among children presenting with different clinical symptoms. The sub-assemblages were identified through PCR-RFLP of the *β-giardin* gene

### Phylogenetic analysis

**Fig. 1:**
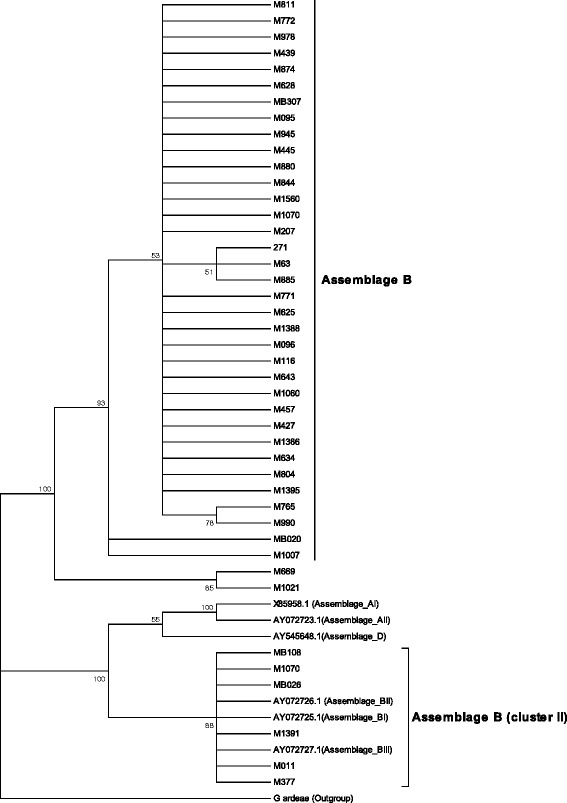
Evolutionary relationships of *G. duodenalis* isolated from selected test samples. The evolutionary history was inferred using the Neighbor-Joining method [44]. The optimal tree with the sum of branch length = 1.10022 is shown. The percentage of replicate trees in which the associated taxa clustered together in the bootstrap test (2000 replicates) are shown next to the branches (Felsenstein et al., 1985). The evolutionary distances were computed using the p-distance method (Nei M et al., 2000) and are in the units of the number of base differences per site. The analysis involved 50 nucleotide sequences. All positions containing gaps and missing data were eliminated. There were a total of 289 positions in the final dataset. Evolutionary analyses were conducted in MEGA5 [20]. The samples are coded according to where they were recruited from and patient number. M1070 refers to Mukuru patient (outpatient) number 1070, MB108 refers to Mbagathi patient (inpatient) number 108

The β-*giardin* locus was amplified in 60 samples of which 41 gave strong DNA products for sequencing. The targeted *β*-giardin sequence fragment was sequenced in thirty two isolates while nine gave short sequences that could not be analyzed. Based on the resulting sequences 30 *G. duodenalis* were categorized as assemblage B, and only two isolates M669 and M1021n were identified as assemblage A. These two isolates had been identified as of mixed infections with sub-assemblages AII and BIII through *GDH* PCR-RFLP analysis. Phylogenetic analysis grouped the isolates into three main clusters namely cluster I which contained assemblages B, with the majority of the isolates clustering within this clade (Fig 1). Cluster II, contained assemblage B with isolates (MB108, M1070, MB026, M1391, M011, M377) clustering with sub assemblages BI, BII, and BIII. Assemblage A (two isolates) clustered distinctly from the reference assemblage A isolates.

### Distribution of Assemblages and sub-assemblages

The distribution of 72 assemblages successfully genotyped among the inpatients was 5 (7 %) and 67 (93 %) from outpatients of which 44 (61 %) were isolated from males and 28 (39 %) from female patients (Table 1). Giardiasis was more prevalent among the children between 13 to 48 months old with 56 (77.8 %) compared to 16 (22.2 %) the 0–12 and 49–60 months old (Table 1). We recorded seven assemblage B infections from HIV infected children. The only assemblage A isolate was from a HIV negative male child. The RFLP of the *GDH* locus is discussed above. All the mixed infection with sub-assemblage BIII and BIV were observed among HIV negative children (Table 2).

## Discussion

*Giardia duodenalis* is among the most common intestinal protozoa and also the most frequent parasitic agent of gastroenteritis especially in the developing countries [21]. This study provides, for the first time in Kenya, data on prevalence and genetic diversity of *G. duodenalis* isolates from children in Kenya.

The genotyping results show that all *Giardia* infections in this population are due to *G. duodenalis* assemblages A and B. This confirms the results of a number of studies performed elsewhere [22]. Distribution of different assemblages differs among and within countries, as surveys in several countries showed a diverse prevalence of assemblages A and B [5]. Here we have shown that children in urban informal settlement in Nairobi, predominantly carry *Giardia* assemblage B, which conforms to reports from several other regions of the world [23–28]. *Giardia* Assemblage B displays high cyst excretion pattern, which in combination with oral-faecal transmission, may contribute to its elevated prevalence rates and broad distribution [29]. On the other hand, studies carried out in Germany, Uganda, Egypt and Portugal, reported a predominance of assemblage A [30–34]. Although both major *G. duodenalis* assemblages A and B have been found in humans throughout the world, their propensity to cause disease might vary.

The predominance of one *G. duodenalis* assemblage over another in a particular area has been attributed to biological as well as geographical factors and, in certain endemic areas; all infections due to *Giardia* in humans appear to involve just one assemblage [35]. The reasons behind the geographic variation in the predominance of the *Giardia* assemblages are still unclear. It may be explained by the difference in the dynamics of transmission. It has been known that assemblage A is most often responsible for zoonotic transmission with wide range of animals acting as reservoir hosts. Although assemblage B is most likely transmitted from human to human, it has been reported in some animals and may represent a zoonotic potential as well [5, 25, 35, 36].

In this study 76 % of the samples amplified successfully with *GDH* primers. Most samples analysed at this locus were identified as assemblage B, except 2 that were assemblage A. These isolates were further identified as sub assemblages AII, and both occurred as mixed infections with sub- assemblage BIII. Results of previous studies elsewhere have shown that humans are mostly infected with AII, although AI is also seen in some studies, while animals are mostly infected with AI, with AII being occasionally reported [22, 25, 37]. The AI sub-assemblage and the B assemblage, regardless of the B sub-assemblages, have a broad host range, including pets, wildlife, and livestock while the AII sub-assemblage is more limited to human subjects [3]. Thus, it is possible the AII infections reported in this study were anthroponotic, while the B infections could have been either zoonotic or anthroponotic. Human to human transmission of *Giardia* infection in the study area could have been exacerbated by the water shortage, and poor sanitary conditions in the slum areas, which has a direct effect on hygiene.

Our study identified both sub-assemblages BIII and BIV in the population, with BIV being commonly isolated. Sprong [11] reported that in Africa, infection with *G. duodenalis* assemblage B, sub-assemblage BIII was more prevalent than infection with sub-assemblage BIV, whereas this differed from findings in North-America where more infections were associated with sub-assemblage BIV, and only few with sub-assemblage BIII, with more balanced distribution being found in Europe and Australia [5]. This however differs with our findings, where BIV is more prevalent. Our study however agrees with findings from Thailand where Assemblage B, sub-assemblage BIV was found to be the most common in preschool children [38].

Occurrence of mixed infections in human cases of giardiasis involving various assemblages appears to be more common than previously thought [16, 17]. *Tpi* assemblage specific primers have proved reliable enough to detect mixed assemblages in the presence of a few copies of the *Giardia* genomes [8, 17, 39]. Co-infection by both *Giardia* assemblage A and B which was observed in 6 cases has been previously reported in Ethiopia and Rwanda [9, 25]. Co-infections with other rare assemblages have also been observed in Ethiopia, where mixed infections with A+ F were reported. In our study mixed infections with sub-assemblage BIII and BIV were frequently observed in 28(56 %) of cases, while AII&BIII was observed in 2 cases. Remarkably, mixtures between BIII and BIV have been previously commonly reported [11]. The occurrence of mixed infections by several assemblages/sub-assemblages of *G. duodenalis* reflects the complex circulation of the parasite in the environment and the exposure of the study population to multiple sources [40].

Phylogenetic analysis of the isolates after bi-directional sequencing of the *β giardin* gene showed that the assemblage B test isolates, formed two clusters. This could be attributed to genetic variation between reference sequences and the test samples, which after comparison of base pair position with the reference B assemblages revealed sequence profile variation within our isolates. A high degree of polymorphism in assemblage B has been observed in other studies [10, 28, 41], and has been further investigated by cloning [42, 43]. This feature has been attributed to mixed subtype infections or allelic sequence divergence, or a combination of both. Assemblage B Kenyan isolates, formed two sub-grouping (Assemblage B, Assemblage B, cluster II). This could be attributed to genetic variation between reference sequences (AY072726.1, AY072725.1). Comparison of base pair position between, reference B assemblages, revealed sequence profile variation within the test isolates from the GenBank.

There was good agreement between assignment of assemblages at all three loci, with assemblage swapping (i.e., different assemblages at different loci in the same isolate) not being observed in any of the isolates. Assemblage swapping has been reported by other investigators [10, 21, 26] and has been attributed to recombination between assemblages or mixed assemblage infection.

## Conclusion

The study provides some preliminary data on assemblage and sub-assemblage distribution of *G. intestinalis* in the country and highlighted that *Giardia* assemblages A and B are prevalent in children in Kenya, with a predominance of assemblage B. These findings suggest that anthroponotic transmission could be a dominant transmission route for giardiasis in Kenya, though there is need to explore the possibility of zoonotic transmission.

## References

[1] Thompson RC, Hopkins RM, Homan WL (2000). Nomenclature and genetic groupings of Giardia infecting mammals. Parasitol Today.

[2] Lasek-Nesselquist E, Welch DM, Sogin ML (2010). The identification of a new Giardia duodenalis assemblage in marine vertebrates and a preliminary analysis of G. duodenalis population biology in marine systems. Int J Parasitol.

[3] Adam RD (2001). Biology of Giardia lamblia. Clin Microbiol Rev.

[4] Monis PT, Caccio SM, Thompson RC (2009). Variation in Giardia: towards a taxonomic revision of the genus. Trends Parasitol.

[5] Feng Y, Xiao L (2011). Zoonotic potential and molecular epidemiology of Giardia species and giardiasis. Clin Microbiol Rev.

[6] Read CM, Monis PT, Thompson RC (2004). Discrimination of all genotypes of Giardia duodenalis at the *glutamate dehydrogenase* locus using PCR-RFLP. Infect Genet Evol.

[7] Traub RJ, Monis PT, Robertson I, Irwin P, Mencke N, Thompson RC (2004). Epidemiological and molecular evidence supports the zoonotic transmission of Giardia among humans and dogs living in the same community. Parasitology.

[8] Foronda P, Bargues MD, Abreu-Acosta N, Periago MV, Valero MA, Valladares B (2008). Identification of genotypes of Giardia intestinalis of human isolates in Egypt. Parasitol Res.

[9] Gelanew T, Lalle M, Hailu A, Pozio E, Caccio SM (2007). Molecular characterization of human isolates of Giardia duodenalis from Ethiopia. Acta Trop.

[10] Caccio SM, Beck R, Lalle M, Marinculic A, Pozio E (2008). Multilocus genotyping of Giardia duodenalis reveals striking differences between assemblages A and B. Int J Parasitol.

[11] Sprong H, Caccio SM, van der Giessen JW (2009). Identification of zoonotic genotypes of Giardia duodenalis. PLoS Negl Trop Dis.

[12] Lalle M, di Frangipane RA, Poppi L, Nobili G, Tonanzi D, Pozio E (2007). A novel Giardia duodenalis assemblage A subtype in fallow deer. J Parasitol.

[13] Monis PT, Mayrhofer G, Andrews RH, Homan WL, Limper L, Ey PL (1996). Molecular genetic analysis of Giardia intestinalis isolates at the *glutamate dehydrogenase* locus. Parasitology.

[14] Haque R, Roy S, Kabir M, Stroup SE, Mondal D, Houpt ER (2005). Giardia assemblage A infection and diarrhea in Bangladesh. J Infect Dis.

[15] Read C, Walters J, Robertson ID, Thompson RC (2002). Correlation between genotype of Giardia duodenalis and diarrhoea. Int J Parasitol.

[16] Geurden T, Geldhof P, Levecke B, Martens C, Berkvens D, Casaert S (2008). Mixed Giardia duodenalis assemblage A and E infections in calves. Int J Parasitol.

[17] Levecke B, Geldhof P, Claerebout E, Dorny P, Vercammen F, Caccio SM (2009). Molecular characterisation of Giardiaduodenalis in captive non-human primates reveals mixed assemblage A and B infections and novel polymorphisms. Int J Parasitol.

[18] Mbae CK, Nokes J, Mulinge E, Nyambura J, Waruru A, Kariuki S (2013). Intestinal parasitic infections in children presenting with diarrhoea in outpatient and inpatient settings in an informal settlement of Nairobi. Kenya. BMC Infect Dis..

[19] Thompson JD, Gibson TJ, Plewniak F, Jeanmougin F, Higgins DG (1997). The CLUSTAL_X windows interface: flexible strategies for multiple sequence alignment aided by quality analysis tools. Nucleic Acids Res.

[20] Tamura K, Peterson D, Peterson N, Stecher G, Nei M, Kumar S (2011). MEGA5: molecular evolutionary genetics analysis using maximum likelihood, evolutionary distance, and maximum parsimony methods. Mol Biol Evol.

[21] Thompson RC, Smith A (2011). Zoonotic enteric protozoa. Vet Parasitol.

[22] Caccio SM, Ryan U (2008). Molecular epidemiology of giardiasis. Mol Biochem Parasitol.

[23] Ignatius R, Gahutu JB, Klotz C, Steininger C, Shyirambere C, Lyng M (2012). High prevalence of Giardia duodenalis Assemblage B infection and association with underweight in Rwandan children. PLoS Negl Trop Dis.

[24] Ankarklev J, Hestvik E, Lebbad M, Lindh J, Kaddu-Mulindwa DH, Andersson JO (2012). Common coinfections of Giardia intestinalis and Helicobacter pylori in non-symptomatic Ugandan children. PLoS Negl Trop Dis.

[25] Lebbad M, Petersson I, Karlsson L, Botero-Kleiven S, Andersson JO, Svenungsson B (2011). Multilocus genotyping of human Giardia isolates suggests limited zoonotic transmission and association between assemblage B and flatulence in children. PLoS Negl Trop Dis.

[26] Breathnach AS, McHugh TD, Butcher PD (2010). Prevalence and clinical correlations of genetic sub-assemblages of Giardia lamblia in an urban setting. Epidemiol Infect.

[27] Yang R, Lee J, Ng J, Ryan U (2010). High prevalence Giardia duodenalis assemblage B and potentially zoonotic sub-assemblages in sporadic human cases in Western Australia. Int J Parasitol.

[28] Lebbad M, Ankarklev J, Tellez A, Leiva B, Andersson JO, Svard S (2008). Dominance of Giardia assemblage B in Leon, Nicaragua. Acta Trop.

[29] Kohli A, Bushen OY, Pinkerton RC, Houpt E, Newman RD, Sears CL (2008). Giardia duodenalis assemblage, clinical presentation and markers of intestinal inflammation in Brazilian children. Trans R Soc Trop Med Hyg.

[30] Karanis P, Ey PL (1998). Characterization of axenic isolates of Giardia intestinalis established from humans and animals in Germany. Parasitol Res.

[31] Yong TS, Park SJ, Hwang UW, Yang HW, Lee KW, Min DY (2000). Genotyping of Giardia lamblia isolates from humans in China and Korea using ribosomal DNA Sequences. J Parasitol.

[32] El-Shazly AM, Mowafy N, Soliman M, El-Bendary M, Morsy AT, Ramadan NI (2004). Egyptian genotyping of Giardia lamblia. J Egypt Soc Parasitol.

[33] Sousa MC, Morais JB, Machado JE, Poiares-da-Silva J (2006). Genotyping of Giardia lamblia human isolates from Portugal by PCR-RFLP and sequencing. J Eukaryot Microbiol.

[34] Johnston AR, Gillespie TR, Rwego IB, McLachlan TL, Kent AD, Goldberg TL (2010). Molecular epidemiology of cross-species Giardia duodenalis transmission in western Uganda. PLoS Negl Trop Dis.

[35] Volotao AC, Costa-Macedo LM, Haddad FS, Brandao A, Peralta JM, Fernandes O (2007). Genotyping of Giardia duodenalis from human and animal samples from Brazil using beta-giardin gene: a phylogenetic analysis. Acta Trop.

[36] van Keulen H, Macechko PT, Wade S, Schaaf S, Wallis PM, Erlandsen SL (2002). Presence of human Giardia in domestic, farm and wild animals, and environmental samples suggests a zoonotic potential for giardiasis. Vet Parasitol.

[37] Xiao L, Fayer R (2008). Molecular characterisation of species and genotypes of Cryptosporidium and Giardia and assessment of zoonotic transmission. Int J Parasitol.

[38] Boontanom P, Mungthin M, Tan-Ariya P, Naaglor T, Leelayoova S (2011). Epidemiology of giardiasis and genotypic characterization of Giardia duodenalis in preschool children of a rural community, central Thailand. Trop Biomed.

[39] Almeida A, Pozio E, Caccio SM (2010). Genotyping of Giardia duodenalis cysts by new real-time PCR assays for detection of mixed infections in human samples. Appl Environ Microbiol.

[40] Smith HV, Caccio SM, Tait A, McLauchlin J, Thompson RC (2006). Tools for investigating the environmental transmission of Cryptosporidium and Giardia infections in humans. Trends Parasitol.

[41] Lalle M, Bruschi F, Castagna B, Campa M, Pozio E, Caccio SM (2009). High genetic polymorphism among Giardia duodenalis isolates from Sahrawi children. Trans R Soc Trop Med Hyg.

[42] Hussein AI, Yamaguchi T, Nakamoto K, Iseki M, Tokoro M (2009). Multiple-subgenotype infections of Giardia intestinalis detected in Palestinian clinical cases using a subcloning approach. Parasitol Int.

[43] Kosuwin R, Putaporntip C, Pattanawong U, Jongwutiwes S (2010). Clonal diversity in Giardia duodenalis isolates from Thailand: evidences for intragenic recombination and purifying selection at the beta giardin locus. Gene.

[44] Saitou N, Nei M (1987). The neighbor-joining method: a new method for reconstructing phylogenetic trees. Mol Biol Evol.

